# Older Adults’ Knowledge and Perceptions of Whole Foods as an Exercise Recovery Strategy

**DOI:** 10.3389/fnut.2021.748882

**Published:** 2021-10-04

**Authors:** Eleanor Jayne Hayes, Antoneta Granic, Christopher Hurst, Lorelle Dismore, Avan A. Sayer, Emma Stevenson

**Affiliations:** ^1^AGE Research Group, Translational and Clinical Research Institute, Newcastle University, Newcastle upon Tyne, United Kingdom; ^2^NIHR Newcastle Biomedical Research Centre, Newcastle upon Tyne Hospitals NHS Foundation Trust and Newcastle University, Newcastle upon Tyne, United Kingdom; ^3^Population Health Sciences Institute, Newcastle University, Newcastle upon Tyne, United Kingdom

**Keywords:** exercise recovery, whole foods, dietary protein, older adults, online survey

## Abstract

Resistance exercise is a widely advocated treatment for improving muscle strength and performance in older adults. Maximizing the benefit of resistance exercise by ensuring optimal recovery is an important aim and studies are now seeking interventions to expedite exercise recovery in older people. A recovery strategy that has acquired considerable interest is the consumption of protein, and more recently, the consumption of protein-rich whole foods. This study aimed to understand the perspectives of community-dwelling older adults, and determine their knowledge of exercise recovery strategies, their preferences for recovery strategies, and their attitudes toward using whole foods, such as milk as a post-exercise recovery aid. Two hundred ninety-one older adults (74 ± 4 years) were recruited to complete a self-administered online survey. A mixed methods approach was used to gather in-depth data from the cohort. Participants were asked to complete a combination of free-text (open-ended) and multiple-choice questions. Content analysis was conducted on responses to open-ended questions through a systematic classification process of coding. The most common recovery strategies reported were heat treatment, rest, and massage. Nutrition was rarely cited as a recovery strategy. Less than 2% of respondents mentioned nutrition, of these, only half mentioned a protein source. Forty-nine percent expressed negative opinions toward recovery supplements (e.g., “waste of money”) compared to 7% expressing positive opinions. Whole foods such as milk, meat, fish, and fruit, were deemed to be a more acceptable recovery strategy than supplements by 80% of respondents. Those that found whole foods to be equally as acceptable (18%), cited efficacy as their main concern, and those that declared whole foods less acceptable (2%) had no common reason. Despite the high acceptability of whole foods, only 35% were aware that these foods could aid recovery. When asked about milk specifically, the majority of older adults (73%) said this would, or might, be an acceptable exercise recovery strategy. Those that found milk an unacceptable recovery strategy (27%) often cited disliking milk or an allergy/intolerance. In conclusion, whilst whole foods represented an acceptable recovery intervention for older adults, the majority were unaware of the potential benefits of nutrition for post-exercise recovery.

## Introduction

Resistance exercise is a widely advocated treatment for improving muscle health in older adults (1). However, despite a considerable body of literature demonstrating the benefits of resistance exercise in older adults (2, 3), the process of recovery from resistance exercise is largely understudied. Similarly, the identification of efficacious and acceptable exercise recovery strategies in an older population has not yet been achieved.

Maximizing the potential benefit of resistance exercise programs by ensuring optimal recovery is an important aim. Indeed, any transient decrements in physical functioning as a result of resistance exercise could be detrimental to an older individual when performing habitual daily activities (4, 5). Similarly, rapid and effective recovery from exercise underpins both improvements in physical performance and body punctuation, Therefore, many studies are now seeking to identify interventions to expedite exercise recovery in older people (6). Acute recovery from resistance exercise is a multifaceted process, and hence, any recovery strategy must consider the underlying physiological processes that occur. These processes include, but are not limited to, increasing rates of muscle protein synthesis and muscle protein breakdown, a coordinated cytokine response, rehydration, and glycogen resynthesis (7, 8). A recovery strategy that has therefore acquired considerable interest is the consumption of protein, and more recently, the consumption of protein-rich whole foods.

The beneficial effects of post-exercise protein supplementation on exercise recovery has been well-documented in younger adults (9, 10). In a recent meta-analysis, post-exercise intake of whey protein, which is rich in the amino acid leucine, was shown to expedite the recovery of muscle function in young adults (10). A potential whole food nutritional intervention that has both a high whey and leucine content is cow’s milk. Semi-skimmed milk contains approximately 3.6 g of protein per 100 ml, of which approximately 80% is whey protein, and the remaining 20% casein. Milk also contains several essential micronutrients such as calcium and sodium, alongside carbohydrates (4.7–5.0 g/100 ml) and varying amounts of fats (0.3–3.7 g/100 ml), and is also approximately isotonic (11). This unique nutritional composition aids rehydration, glycogen synthesis, and restores energy balance, alongside optimizing muscle protein turnover and reducing muscle soreness (11). Hence, milk could be considered an optimal post-exercise recovery beverage. Indeed, it has already been shown that the ingestion of milk following muscle damaging exercise can attenuate muscle soreness and decrements in physical performance in younger adults (12–14). As a readily available and cheap food source, cow’s milk may therefore potentially be a useful recovery strategy for older adults. However, despite evidence for the role of nutrition in enhancing exercise recovery in both younger (14, 15) and older (6) adults, current evidence of its acceptability amongst older adults is scarce—only one recent study has explored the feasibility and acceptability of milk as a post-exercise beverage (15). Similarly, the authors are unaware of any literature that has sought to define the perspectives of older adults, or determine their knowledge of, exercise recovery strategies, or exercise recovery supplements. To inform the direction of future research in this area, it is important that researchers and practitioners alike understand what exercise recovery strategies are currently used by older adults, their attitudes toward recovery supplements, and the acceptability of whole foods as recovery interventions. As such, this study aimed to understand the perspectives of community-dwelling older adults, and determine their current knowledge of exercise recovery strategies, their preferences for recovery strategies, and their attitudes toward using whole foods, such as milk as a post-exercise recovery aid.

## Materials and Methods

### Design and Procedures

**Figure 1:**
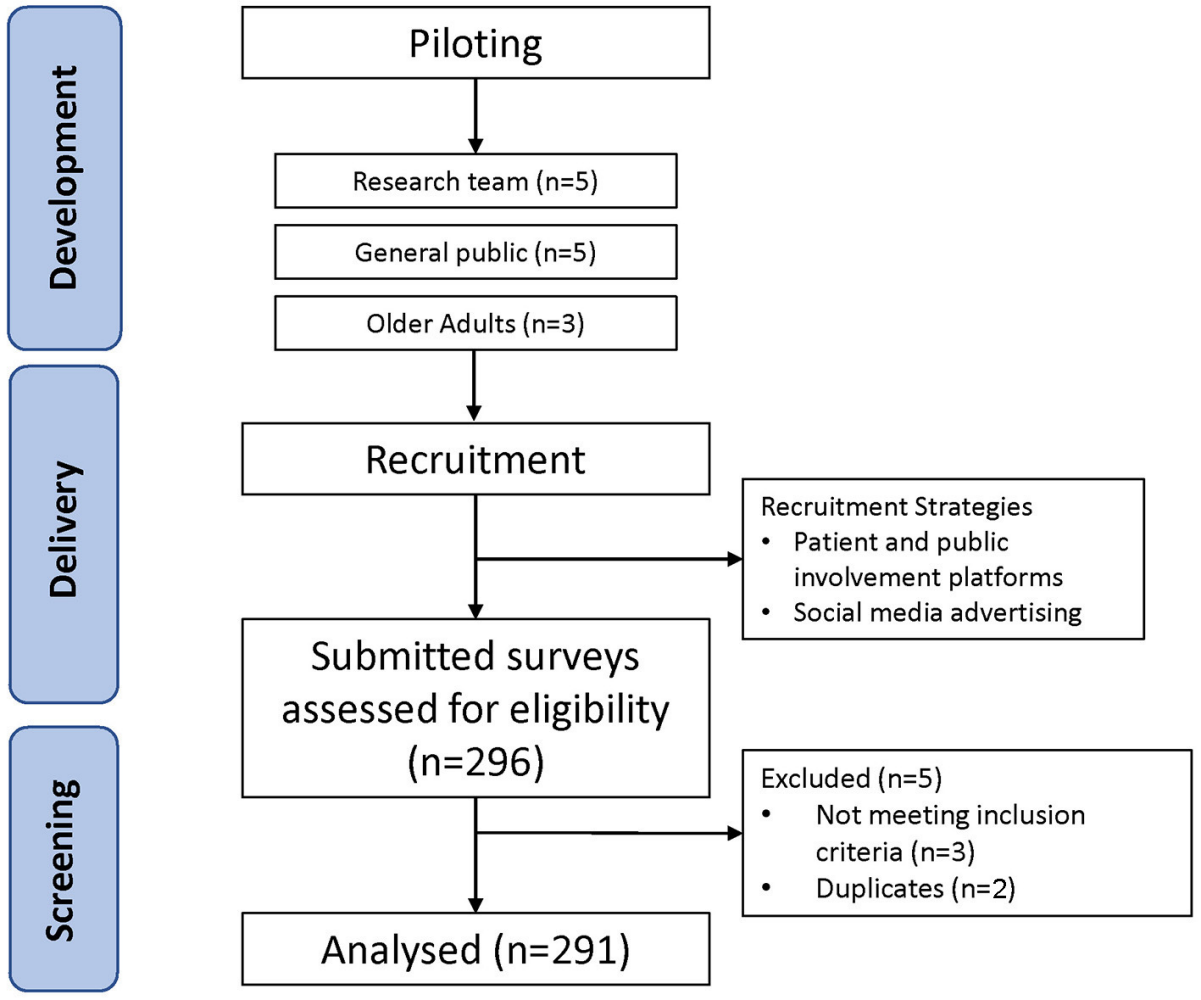
Schematic of methodology.

An online survey was developed that sought to understand older adults’ knowledge in several categories. This was a broad survey, and hence, only one section of the data is reported in this paper. This section included (1) knowledge of exercise recovery strategies, (2) preferences for recovery strategies, and (3) attitudes toward using whole foods, such as milk as a post-exercise recovery aid. Questions pertaining to participant characteristics were included at the start of the survey. In total, the survey featured 32 questions (Appendix A in Supplementary Material). This paper focusses on the findings from question 22 onwards. The survey was administered over a 7-week period during July–August 2020 via *OnlineSurveys* (www.onlinesurveys.ac.uk), and was only available in English. An overview of the methodology can be found in Figure 1.

This survey used a mixed-methods approach. Participants were asked to complete a combination of free-text (open ended) and multiple-choice questions. A vignette was provided within the section “attitudes toward exercise recovery interventions” to help with contextualization of the following questions. For some multiple-choice questions, participants were prompted to answer further questions, or give a free-text response to provide additional information. For example, if participants answered “Yes” to the question “Have you ever purchased an exercise recovery supplement for yourself?” they were directed to answer the follow-up question “What supplement(s) did you purchase?”

### Piloting

In the first instance, the survey was sent electronically to all members of the research team to ensure attainment of research aims and some minor amendments to wording and answer order were made. To assess the clarity of the questions and the usability of the survey platform, the survey was piloted by a small sample (~five individuals) who provided feedback and submitted responses to the survey. This data was exported to ensure that the questions and requested answer type were correctly formatted. Finally, the survey was sent to three older adults for further feedback. Only minor amendments were made following this process.

### Ethics

Ethical approval for this study was granted by Newcastle University Ethics Committee (Ref: 3648/2020). Before completing the survey, participants were given a brief overview of the purpose and aims of the study and were provided with contact details of the research team should they have had any questions. Participants were informed that by submitting their responses they were providing consent to participate in the questionnaire. Participants were able to withdraw at any time before submitting their responses by simply closing the tab on their web browser. If participants withdrew by closing the tab, no data was collected, but they could choose to participate again at any time by re-opening the survey on their browser.

### Participants

Participants were recruited through social media advertising (Facebook and Twitter), and patient and public involvement platforms (www.voice-global.org). Any individual over the age of 70 years with internet access could complete the survey—no other inclusion or exclusion criteria were applied.

### Data Analysis

Three pre-defined categories were established to meet the aims of the project including; knowledge of exercise recovery strategies, attitudes toward exercise recovery supplements, and attitudes toward cow’s milk as an exercise recovery strategy. A convergent parallel design was used to analyse both the quantitative and qualitative data. Answers given to multiple choice questions are reported as a fraction followed by a percentage. Manifest content analysis was conducted on responses to open-ended questions through a systematic classification process of coding to quantify the identification of words or concepts (16). Data were read and re-read to ensure familiarization and understanding, and interesting aspects of the data were highlighted that captured key concepts in relation to the pre-defined categories. Where more than one code could be derived from a singular free-text answer, codes are reported as a frequency. Where codes are mutually exclusive, data are reported as a frequency followed by a percentage. All percentages are rounded to the nearest whole integer. Where an answer could not be designated to a code, for example, if there were no other similar answers or it was not deemed to be answering the question, it was denoted as “‘unassigned.” If data are missing, the results are reported as a frequency and/or percentage of the sum of responses for that specific question, as opposed to the total number of participants. Similarly, if the question was such that an answer could be assigned more than one code (for example, if an answer mentioned several recovery strategies), then results are reported as frequencies only, and do not include a percentage.

## Results

### Participant Characteristics

At the time of survey closure, 296 responses to the survey had been submitted. After initial screening, five responses were removed (Duplicate participant *n* = 2; Under 70 years of age *n* = 3) leaving 291 older adults (74 ± 4 years; mean ± SD) included in the final analysis. Participants were largely independent, with 95% of respondents requiring no help to perform any activities of daily living, and 89% self-reporting at least “good” physical health. Twenty-three percent of participants that reported their gender were male, and 77% were female. Participants were also moderately active. Forty-two percent reported that they regularly participate in aerobic training (e.g., cycling, jogging, spinning classes, dancing, swimming), and 25% reported participating in resistance exercise. Fewer than 32% of respondents said that they perform <1 h of physical activity per day.

### Knowledge of Exercise Recovery Strategies

The most common recovery strategy that older adults’ reported using was heat treatment, including both hot baths/showers and cold treatments (*n* = 107). Other common exercise recovery strategies included rest (*n* = 77), stretching (*n* = 41), gentle exercise (*n* = 32), painkillers (*n* = 31), massage (*n* = 28), and topical ointments (*n* = 22) (Table 1). The use of nutrition as a recovery strategy was mentioned by only five (<2%) individuals, of these, only two reported using a high protein food.

**Table 1:** Exercise recovery strategies reported by older adults^a^.

**Code**	***n***	**Examples**
Heat treatment	107	• Used to take a long soak in a warm bath after a day walking
		• Hot water bottle
		• Use cold compress if necessary
		• Cold water shower over the muscles
Rest	77	• Have a day’s rest then exercise muscles again. To get rid of lactic acid
		• Rested for a while until it eased
Stretching	41	• Stretched the area if possible
		• Gentle yoga
Gentle exercise	32	• Keep going with daily activities but avoid strenuous exercise for a few days and gradually increase
		• Took gentle walking exercise. Tried not to sit for long periods
Painkillers	31	• Two paracetamol often relieves the soreness and allows me to continue exercising
		• Take ibuprofen tablets or topical gel
Massage	28	• Massage the muscles
		• Used foam roller
Topical ointments	22	• Used over the counter pain relief rubs
		• 2 × Arnica 30c every 2 h
Drank water	16	• Drink plenty of water
Nothing	23	• Nothing, just carried on as normal
		• Usually gone in a couple of days without any intervention
Other	9	• A cup of coffee
		• I warmed up before exercise and stretched the muscles afterwards
		• I know about milk assisting recovery, but usually I don’t do anything special
Change exercise routine	5	• Made sure I didn’t push my body all out once start off slowly

a *“You may have experienced muscle soreness in the past after heavy gardening or DIY, jogging or exercising in a gym. If so, what did you do to ease muscle soreness and improve exercise recovery?” (291 responses)*.

### Attitudes Toward Exercise Recovery Supplements

Ninety-five percent of respondents had never bought an exercise recovery supplement, and <1% had ever bought a protein supplement. Only 7% of older adults responded positively when asked their views on exercise recovery supplements, and 49% expressed negative views such as “waste of money” (Table 2). Eighty percent of respondents said that the use of whole foods (e.g., berries, fruit, meat, milk, and fish) was more acceptable than supplements as an exercise recovery strategy for reasons such as “food is more natural” and it being a “healthy diet” (Table 3). Those that were unsure if whole foods were more acceptable (18%) generally reported concerns of efficacy (Table 3), and those that found whole foods less acceptable (2%) generally referenced allergies/intolerances or ethical concerns. Despite whole foods being deemed more acceptable than supplements by a large majority of older adults, only 35% were aware that these foods could aid recovery.

**Table 2:** Older adults’ views on exercise recovery supplements^*a*^.

**Code**	***n*** (**%)**	**Example**
**Positive**	20 (7)	• Would be willing to give it a try
		• Some are beneficial to well-being
**Neutral**	43 (15)	• I’d prefer not to take any myself but think it’s ok to do so
		• So-so some work, some don’t
		• Proof they worked and how effective their use was to other older people, would be my view
**Negative**	144 (49)	• I would always be reluctant to take a supplement
		• Waste of money
		• Rubbish-a market encouragement to make money
**No Opinion**	84 (29)	• I know nothing about them
		• No idea, didn’t know there was such a thing

a *“What are your views on exercise recovery supplements for older adults?” (291 responses)*.

**Table 3:** Older adults’ reasons for the acceptability of whole foods for exercise recovery compared to supplements^a^.

**Code**	***n***	**Examples**
**More Acceptable**		
Food is more natural	92	• I would rather rely on natural food than supplements
		• It seems like a more natural process
It is a healthy diet	47	• Eating those food items would be good for me, regardless of the reason for taking them.
Dislike of supplements	24	• I don’t like taking supplements or medication if I can avoid them
		• I am not a great pill popper so doing things through diet makes more sense.
I eat these foods already	19	• They are what I would eat normally anyway
Cost/Accessibility	11	• They carry no additional cost and no profit for charlatan’s
		• Easier to implement and probably cheaper
Know what is in food	10	• I prefer to know what I am eating
		• Don’t like not knowing what supplements are made of
Enjoyment of food	9	• More pleasant to consume
*Unassigned*	24	
**Equally Acceptable**		
Unsure of efficacy	12	• Will it seriously make a difference?
Already eat these foods	6	• I try to have a diet including such items already
Open to anything	5	• I’m quite flexible in other views to relieving post exercise pain
Food is a better option	5	• Fresh fruit and meat are always a better option
Supplements are easier	3	• Sometimes supplements are easier to take
*Unassigned*	11	

a *“Are whole foods more or less acceptable to you than supplements as an exercise recovery intervention? What is your reason for this?” (291 responses)*.

### Attitudes Toward Cow’s Milk as an Exercise Recovery Strategy

**Figure 2:**
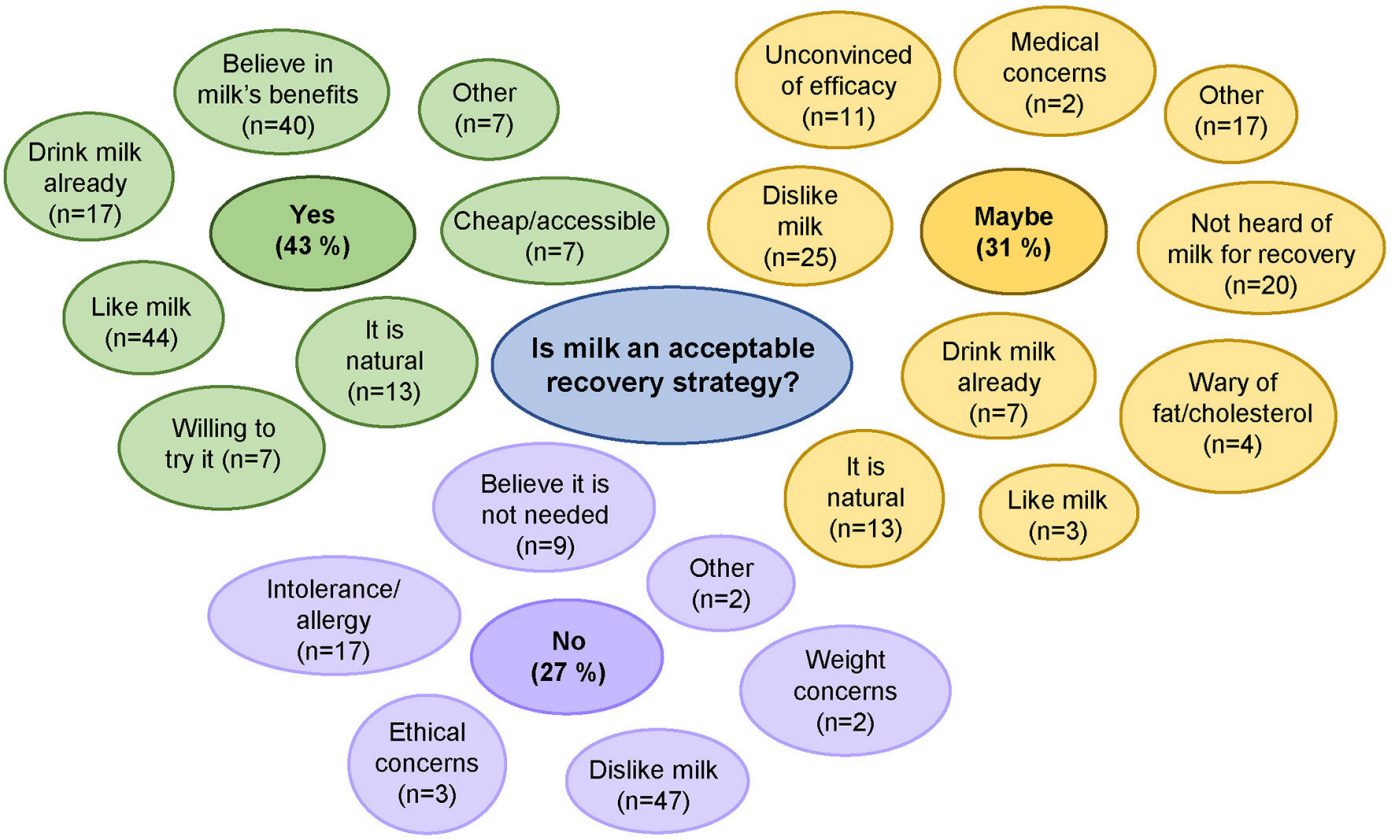
Participant responses to; “We are specifically interested in milk as an exercise recovery beverage in older adults. Would this be an acceptable strategy to you? Please explain your answer?”

When asked specifically if they thought milk was an acceptable recovery strategy, 43% said “yes” due to already liking milk, or believing in milk’s health benefits (Figure 2); for example, one participant stated “Milk is a protein and would help building the damaged muscle” (Supplementary Table 4). Those that said milk was not acceptable (27%) generally disliked milk or had allergies/intolerances (Figure 2). Thirty-one percent said that milk “may be” be an acceptable recovery strategy, with the most common reasons for hesitancy being that they disliked milk (*n* = 25), or had not heard of milk as a recovery aid before (*n* = 20) (Figure 2). Others that were unsure were also unconvinced of the efficacy of milk for recovery (e.g., “If you could show me the science behind this I would be willing to try”). Just 46% of respondents said they thought they would be able to drink the suggested 500 ml of milk, and only 44% said they would be willing to drink 500 ml of milk after exercise.

## Discussion

**Figure 3:**
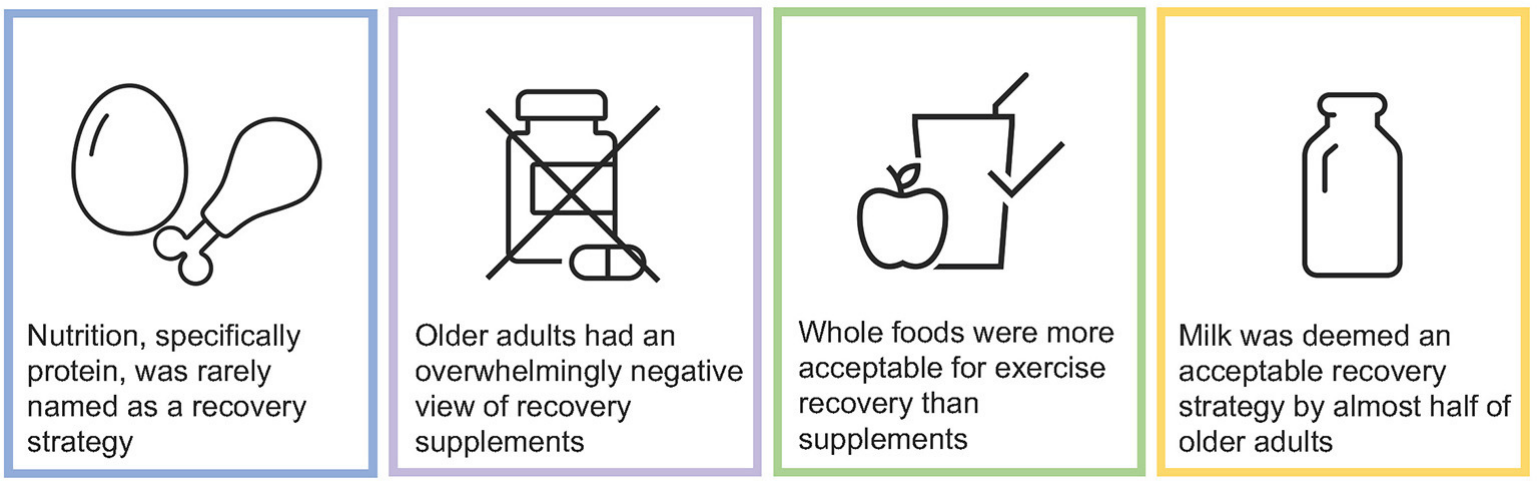
Summary of main findings.

This study aimed to understand the perspectives of community-dwelling older adults, and determine their current knowledge of exercise recovery strategies, their preferences for recovery strategies, and their attitudes toward using whole foods, such as milk as a post-exercise recovery aid. To our knowledge this is the first study to explore these concepts in adults over 70 years of age using an online survey platform. Our main finding is that knowledge of nutritional strategies for exercise recovery was poor amongst older individuals, and despite not being aware of their benefits, whole foods were considered to be more acceptable than supplements for exercise recovery (see Figure 3).

### Knowledge of Exercise Recovery Strategies

To determine what older adults currently know about exercise recovery strategies, participants were asked what exercise recovery strategies they had used in the past. The most common recovery strategy was the use of heat treatment by approximately a third of older adults, which included both hot (e.g., using a hot water bottle) and cold (e.g., using a cold compress) treatments. As the use of heat treatments is widely used for muscular complaints and injuries, this is perhaps unsurprising. Interestingly, rest was only the second most common stated recovery strategy, but this could be due to rest being a passive strategy, rather than being thought of as an active recovery strategy. Gentle exercise, stretching, massage, and topical ointments such as heat rub, were also popular. Drinking water was mentioned on 16 occasions as a recovery strategy. However, other than drinking water, nutrition was mentioned by only five individuals. Considering the research to date demonstrating the potential effectiveness of nutrition as a recovery aid for both younger (8, 17) and older adults (6), it is surprising that few older adults in this survey reported nutrition as a strategy to help muscle soreness. This suggests a discord between the knowledge of researchers and older members of the general public population, and displays a need for improved education and patient and public involvement when identifying suitable exercise recovery strategies for older adults.

Similarly, protein as an exercise recovery aid was mentioned by only two individuals (<1% of the sample). The low reported incidence of dietary protein as a recovery aid in the current study may have been exaggerated as a result of a general lack of understanding of exercise recovery and muscle soreness as a whole. However, our findings may demonstrate a lack of awareness, or understanding, of the benefits of dietary protein amongst older adults. Although not directly investigating protein for exercise recovery, a recent study has also found poor knowledge of dietary protein amongst 1,825 community-dwelling European older adults. Using an online survey, it was determined that 35.3% of the sample did not know what dietary protein was, and low protein knowledge was observed in 902 (49.4%) participants of the total study sample (18). Of more relevance to the current study is that amongst individuals with low protein knowledge, only 65% responded correctly to the statement that “You need protein in the diet for repairing bones and muscles.” Similarly, a recent feasibility study has shown that giving older adults dietary advice and protein rich food products increased their dietary protein intake over 4 weeks, and most reported that they would continue following the advice on cessation of the study (19). If these results are translatable to a post-exercise setting, it is possible that there would be good compliance with protein-rich foods as an exercise recovery strategy. As a whole, these findings demonstrate a clear need to educate older adults on the benefits of protein, both for health and as an exercise recovery strategy. However, this is not necessarily exclusive to dietary protein. As mentioned previously, very few individuals named nutrition as an exercise recovery strategy and hence, education of several areas of nutrition may be warranted for older adults.

### Attitudes Toward Exercise Recovery Supplements

Exercise recovery supplements have been shown to be efficacious (20) and are widely used (21, 22) in younger adults. However, the vast majority of older adults in this study had never bought an exercise recovery supplement, and <1% had ever bought a protein supplement. Given the previous finding that older adults’ knowledge of dietary protein for exercise recovery is limited, it is perhaps unsurprising that so few had purchased exercise recovery supplements. Older adults’ views on exercise recovery supplements is likely also a factor in how few had purchased a supplement in the past. Indeed, only 7% of older adults in this sample expressed a positive opinion of exercise recovery supplements, and almost half voiced a negative opinion (e.g., “*Rubbish—A market encouragement to make money*”). It is therefore unlikely that there would be good compliance to any recommended exercise recovery supplement for older adults. Instead, research may wish to focus on the use of commonplace foods that older adults are familiar with, and can access easily at their supermarket or food supplier.

In support of this, the current study found evidence that whole foods (e.g., berries, fruit, meat, milk, and fish) were a more acceptable recovery strategy than supplements by a large majority (80%) of older adults, despite over a third of older adults not being aware that these foods could aid recovery. The most common reason for this is that food was considered more natural or healthy. However, another common reason for finding whole foods more acceptable was simply a strong dislike of supplements. As mentioned by a number of participants, a whole food approach is also often cheaper than supplements, and hence could be more accessible to a greater proportion of older adults.

Emerging literature also suggests that a food-first approach could be more effective in enhancing post-exercise muscle remodeling when compared to isolated protein supplements in younger adults (23). This is because whole foods often contain other non-protein nutritive components (e.g., carbohydrates, lipids, micronutrients) that may interact to increase muscle protein synthesis rates beyond those expected from isolated proteins alone (23). Interestingly, such foods that may be useful for exercise recovery in older adults (e.g., meats, fish, eggs, legumes, nuts, and dairy) have also been shown to be myoprotective (24, 25), and so their increased consumption would likely complement resistance exercise to further preserve muscle mass and strength. Therefore, future research aiming to identify exercise recovery strategies for older adults could focus on a food-based approach to ensure uptake and adherence, whilst also considering the overall effectiveness of the food-stuff in a post-exercise setting.

### Attitudes Toward Cow’s Milk as an Exercise Recovery Strategy

The high-protein content of milk (3.6 g/100 ml) alongside its nutritional composition of carbohydrates, fats, and micronutrients suggests it could be useful for aiding exercise recovery (11). Indeed, milk has previously been shown to be an effective exercise recovery strategy in younger adults (14, 26), and has been shown to be beneficial for skeletal muscle health (25), but its acceptability to older adults is uncertain (15, 27). We explored older adult’s attitudes toward milk as a high-protein exercise recovery strategy. In our survey, nearly half of older adults considered milk to be an acceptable recovery strategy, whilst approximately a quarter of older adults thought milk was unacceptable, and gave reasons such as dislike of milk, allergies, intolerances, and ethical concerns. Those that thought milk might be acceptable either disliked milk by itself, or had not heard of the benefits of milk as a post-exercise recovery strategy. This suggests that milk may be more acceptable to a larger proportion of older adults if they were educated on the benefits of milk, but there is currently no evidence to support this.

We have identified one recent study which explored older adults’ attitudes and barriers to engaging in a resistance training program and consuming a recovery drink (bovine milk) after exercise (27). The study conducted semi-structured interviews after a 6-week exercise and nutrition intervention aiming to understand older adults’ barriers and motivations for engagement. The study concluded that older adults considered milk to be an acceptable post-exercise recovery drink. Only one participant struggled with the volume of milk (2 × 500 ml), but overall post the taste and volume of liquid were viewed as acceptable. Of interest is that some participants did not think they would find milk acceptable before the intervention began, but began to look forward to consuming their drink (27). The authors concede that there is currently no evidence examining the efficacy of cow’s milk for post-exercise recovery in older adults, but there is a number of studies investigating its use in younger adults (11, 13, 14), and for enhancing muscle protein turnover (28, 29). However, data from a pilot study of community-dwelling older adults suggests that cow’s milk is both an acceptable and feasible post-exercise intervention for this group (15). (15) reported compliance to consuming 2 × 500 ml of milk after resistance exercise twice per week for 6 weeks of 97.1 and 98.3% for whole milk and skimmed milk, respectively, in a group of 29 older adults. Additionally, no participants reported finding the milk intervention difficult, or saw the volume of milk as a barrier. Only two participants reported minor changes to their usual diet as a result of the milk intervention (15). This suggests that should milk be found to be beneficial in older adults, it would be a suitable and accepted strategy for post-exercise recovery. It should be recognized that this study had a small sample size, and participation was likely dependent on the individuals having a positive predisposition toward milk consumption, and hence, the acceptability of this intervention amongst the general population is still unclear. This is promising for milk as an exercise recovery strategy for older adults, but further research must first be conducted to determine the efficacy of milk for recovery in an older population, and to explore other potential high-protein recovery strategies that may be more acceptable.

## Strengths and Limitations

To our knowledge, this is the first study that has attempted to understand older adults’ knowledge of, and attitudes toward, exercise recovery strategies. The results of this study will help to direct future research to identify effective and acceptable exercise recovery strategies for older adults. The use of an online platform to gather these data allowed a high number of participants to be recruited from across the United Kingdom.

However, this study has several limitations. A main concern of the current methodology is that the questionnaire was not validated, and it is therefore possible that some of the questions posed lead to a bias in the response of participants or did not gather the data that was intended. Additionally, some groups, likely those of lower socio-economic status, minority ethnic groups, or the very old, may not have been represented due to a lack of internet access. Unfortunately, to limit participant burden, detailed demographic information and participant characteristics were not collected, and hence, it is not understood to whom the results of the current study are most relevant. The literature would benefit from a similar study that uses semi-structured interviews to address our aims, as this will allow researchers to probe for further information, which was not possible with our survey. Interviews conducted within the community may also allow access to individuals whose views have not been captured by the current study.

## Summary of Findings

Older adult’s knowledge of exercise recovery was limited, and there is a clear need for education, especially concerning the role of nutrition for aiding exercise recovery. Older adults were much more accepting of whole foods as a recovery strategy than supplements, although knowledge of their benefits is poor. More work needs to be done to (a) provide education on exercise recovery foods, (b) identify other high protein, acceptable strategies for older adults other than milk, and (c) identify whole foods to aid recovery for those that cannot consume animal-based products.

## Conclusion

In conclusion, whilst whole foods represented an acceptable exercise recovery intervention for community-dwelling older adults, the majority were unaware of the potential benefits of nutrition for post-exercise recovery. Older adults may benefit from education about beneficial interventions such as nutrition for exercise recovery, and further research should be conducted to determine the efficacy of whole foods such as milk for improving exercise recovery.

## Data Availability Statement

The raw data supporting the conclusions of this article will be made available by the authors, without undue reservation.

## Ethics Statement

Ethical review and approval was not required for the study on human participants in accordance with the local legislation and institutional requirements. The patients/participants provided their written informed consent to participate in this study.

## Author Contributions

EH, AG, CH, LD, ES, and AS contributed to the conception and development of the survey. EH, AG, and ES were responsible for the piloting of the survey. EH distributed the survey and completed data analysis. EH authored the first draft of the manuscript. All authors contributed to manuscript revision, read, and approved the submitted version.

## Funding

This project is funded by the Medical Research Council Versus Arthritis Centre for Integrated Research into Musculoskeletal Ageing CIMA Ph.D. Studentship (EH) and supported by Newcastle University (ES).

## Conflict of Interest

The authors declare that the research was conducted in the absence of any commercial or financial relationships that could be construed as a potential conflict of interest.

## Publisher's Note

All claims expressed in this article are solely those of the authors and do not necessarily represent those of their affiliated organizations, or those of the publisher, the editors and the reviewers. Any product that may be evaluated in this article, or claim that may be made by its manufacturer, is not guaranteed or endorsed by the publisher.
