# Supplementary material for: Older Adults’ Knowledge and Perceptions of Whole Foods as an Exercise Recovery Strategy
Source: Front Nutr. 2021 Oct 4;8:748882. doi: 10.3389/fnut.2021.748882 (PMC8520979; doi:10.3389/fnut.2021.748882)
Supplement: Supplementary file 1 [file Table_1.DOCX]

**Supplementary Materials Appendix A**

**Older Adults and Exercise Recovery Survey (70+ years)**

**Page 1: Introduction**

THANK YOU FOR TAKING THE TIME TO VISIT OUR SURVEY

Before you begin we would like to thank you for taking the time to complete this survey. The data collected from this survey will be used in research aiming to prevent muscle loss with advancing age using exercise. For this reason, we are asking only those over 70 years of age to complete this survey.

This survey should take approximately 10-15 minutes to complete. Please do not skip questions as everything you write is useful. Answer every question to the best of your knowledge, there are no wrong answers! The questions will appear in sections, please ensure you complete all the sections. For data analysis purposes, some answer boxes are limited to just a few words. It is up to you if you wish to participate in this survey. You can withdraw from this survey at any time simply by closing the browser web page. Unfortunately, once you have submitted your survey response, we are unable to remove anonymised data from our records. By submitting your responses to the survey you are providing your consent to participate in this questionnaire and for researchers to access your responses. All data will be anonymised before use in any publication.

At the end of the survey you will be asked if you wish to provide your contact information for future research. You are under no obligation to provide this information. If you do not wish to share this information please leave the answer boxes blank.

If you have any queries regarding the completion of this survey please contact:

e.j.hayes2@newcastle.ac.uk

**Page 2: GDPR Regulations**

Newcastle University will be using information from you in order to undertake this research study and will act as the data controller for this study. This means that Newcastle University is responsible for looking after your information and using it properly. When we use personally identifiable information from people who have agreed to take part in research, we ensure that it is in the public interest. Your rights to access, change or move your information are limited, as

Newcastle University needs to manage your information in specific ways in order for the research to be reliable and accurate. If you withdraw from the study, Newcastle University will keep the information about you that has already been obtained. To safeguard your rights, the minimum personally-identifiable information will be used. You can find out more about how Newcastle University uses your information at https://www.ncl.ac.uk/data.protection/dataprotectionpolicy/privacynotice/ and/or by contacting Newcastle University’s Data Protection Officer (Maureen Wilkinson, rec-man@ncl.ac.uk).

We will use your name and contact details (telephone number, email address) to contact you about the research study if you choose to provide this information. We will use date of birth in order to understand the demographics of our respondents. Individuals at Newcastle University may look at your research data to check the accuracy of the research study. The only individuals at Newcastle University who will have access to information that identifies you will be individuals who need to contact you if you indicated you would be interested in future research.

If you agree to take part in the research study, information provided by you may be shared with researchers running other research studies at Newcastle University and in other organisations.

Your information will only be used by organisations and researchers to conduct research.

**Page 3: Age, Gender, and Physical Capability**

In this section, we will gather data about your age and general physical health

1. What is your date of birth? Dates need to be in the format 'DD/MM/YYYY', for example 27/03/1980.

2. What is your gender?

- Male
- Female
- I'd prefer not to say
- Other

2.a. If you selected Other, please specify:

3. Are you able to walk around the house and indoors?

- Independent (but may use aid e.g. stick)
- Walk with help of one person (physical, verbal or supervision)
- Wheelchair independent
- Immobile

4. Are you able to walk up and down the stairs?

- Able unassisted
- Need help (physical, verbal, carrying aid)
- Unable

5. Please select an answer for each row. Are you able to...

(Please don't select more than 1 answer(s) per row. Please select at least 6 answer(s).)

|  | Independent  (I can do  everything  myself) | I need  some help  from  another  person | I am  dependent  on another  person to  help me |
| --- | --- | --- | --- |
| ...feed yourself? |  |  |  |
| ...groom yourself (hair, teeth, shaving etc.)? |  |  |  |
| ...dress yourself? |  |  |  |
| ...transfer from bed to chair and back? |  |  |  |
| ...use the toilet? |  |  |  |
| ...bathe yourself? |  |  |  |

6. In general, would you say that your physical health is...

- Excellent
- Very good
- Good
- Fair
- Poor

7. In general, would you say that your mental health is...

- Excellent
- Very good
- Good
- Fair
- Poor

**Page 4: Physical Activity Status**

In this section, we will gather data on your current physical activity levels

8. On average, how much time in a day do you spend doing physical activities (e.g. going for a walk, jogging, gardening, housework, DIY, sports activities, stretching)?

- I do not do any physical activities
- Less than 30 minutes
- 30 minutes to 1 hour
- 1 to 2 hours
- 2 to 3 hours
- 3 to 4 hours
- More than 4 hours

9. On average, how much time in a day are you sedentary (e.g. sitting watching TV, reading, computer, sitting outside) excluding sleeping time?

- Less than 2 hours
- 2 to 4 hours
- 4 to 6 hours
- 6 to 8 hours
- 8 to 10 hours
- More than 10 hours

10. Do you participate in any aerobic exercise (e.g. cycling, jogging, spinning classes, dancing, swimming)?

- Yes
- No

10.a. How often do you do this exercise or a combination of different aerobic exercises?

- Once per week
- Twice per week
- Three to four times per week
- Almost every day
- Twice per day
- Not applicable

10.a.i. On average, what intensity is this exercise? Very light (e.g. stretching)

- Light (e.g. slow dancing, boules)
- Moderate (e.g. cycling slowly, walking continuously)
- High (e.g. cycling at moderate pace, mountain hiking)
- Very high (e.g. running)

Resistance training activities are defined as 'any physical activity which produces a muscle contraction against an external force'. This type of exercise includes weight lifting, resistance bands, body weight squats, the use of dumbbells, and any other similar activity.

11. Do you currently participate in resistance training activities (e.g. weight lifting, body weight exercises, resistance band exercises)?

- Yes
- No

11.a. How often do you do this exercise or a combination of different resistance training exercises?

- Once per week
- Twice per week
- Three to four times per week
- Almost every day
- Twice per day
- Not applicable

**Page 5: Barriers and Motivators for Resistance Training Participation**

In this section, we will gather data on your barriers and motivations for participating in resistance training activities.

12. What are the motivators that may encourage you to participate in resistance training activities (e.g. weight lifting, body-weight exercises, resistance band exercises)?

- *(free-text response)*

13. What are the barriers that may prevent you from participating in resistance training (e.g. weight lifting, body-weight exercises, resistance band exercises)?

- *(free-text response)*

14. If you have recently started resistance training, what encouraged you to do so? (Optional)

- *(free-text response)*

15. If you have performed resistance training in the past but no longer do so, what were your reasons for stopping? (Optional)

- *(free-text response)*

16. Assuming you have been given the appropriate equipment and instructions, in what environment would you feel most comfortable performing resistance training?

- Local gym
- Community centre
- Outdoor space
- Home
- Other

16.a. If you selected Other, please specify:

- *(free-text response)*

**Page 6: Knowledge of Exercise Recovery**

Roy has recently begun lifting weights in his local gym to try and improve his leg strength after having a fall. During his last session he decided to challenge himself and lifted heavier weights than usual. Once he had finished his session he drank a glass of milk and made his way home. The next day, Roy experienced soreness in his legs when he stood up from his chair or walked up stairs. This pain got worse over the next day, and then slowly began to subside. Once the pain had gone, Roy felt happy to go to the gym again.

17. Would you expect Roy's muscles to be sore in the days following resistance training?

- Yes
- No

This next session is designed to help us understand what older adults already know about exercise recovery. Please do not skip questions if you do not know the answer, as discovering what is not known is just as important to us.

18. Have you ever heard of the term ‘post-exercise muscle damage’?

- Yes
- No

18.a. If yes, in your own words, can you describe what you understand by the term ‘post-exercise muscle damage’? (Optional)

- *(free-text response)*

19. Have you ever heard the term 'delayed-onset muscle soreness' (DOMS)?

- Yes
- No

19.a. If yes, in your own words, can you describe what you understand by the term 'delayed-onset muscle soreness' (DOMS)? (Optional)

- *(free-text response)*

Muscle soreness can occur as a result of exercise your body finds challenging. It is often described as an aching or burning in the muscles in the hours or days following exercise. For example, Roy experienced pain in his leg muscles when walking up the stairs the day after performing lower body weights.

20. If you were experiencing delayed-onset muscle soreness (DOMS), do you think this would discourage you from completing your usual daily activities (e.g. gardening, cleaning, walking)?

- Yes
- No
- To some extent

21. If you were experiencing delayed-onset muscle soreness (DOMS), do you think this would discourage you from participating in further exercise until the pain had subsided?

- Yes
- No
- To some extent

**Page 7: Attitudes Towards Recovery Interventions**

After Roy had been lifting weights in the gym he drank a glass of cold milk to rehydrate. This is because he has heard that milk has proteins and other nutrients that may help his muscles to recover quicker and prevent muscle soreness.

22. You may have experienced muscle soreness in the past after heavy gardening or DIY, jogging or exercising in a gym. If so, what did you do to ease muscle soreness and improve exercise recovery?

- *(free-text response)*

23. What other recovery strategies can you describe that might reduce muscle soreness that you have not personally used?

- *(free-text response)*

24. This question focuses on exercise recovery supplements specifically. An exercise recovery supplement is specially designed to promote exercise recovery and often comes in the form of food, drink, powders or tablets. Have you ever purchased an exercise recovery supplement for yourself?

- Yes
- No

24.a. What supplement(s) did you purchase?

- *(free-text response)*

25. What are your views on exercise recovery supplements for older adults?

- *(free-text response)*

26. Another strategy to aid exercise recovery and reduce muscle soreness is the consumption of certain whole foods. This can include, but is not limited to; berries, fruit, meat, milk, and fish. Are these options more or less acceptable to you than supplements as an exercise recovery intervention?

- More acceptable
- Less acceptable
- The same

26.a. What is your reason for this?

- *(free-text response)*

26.b. Were you aware that these types of food could improve exercise recovery?

- Yes
- No

27. We are specifically interested in milk as an exercise recovery beverage in older adults. Would this be an acceptable strategy to you?

- Yes
- No
- Maybe

27.a. Please explain your answer?

- *(free-text response)*

28. Research suggests that drinking 500 mL (approximately one pint) of milk after exercise could be effective for improving exercise recovery. Do you think you could drink this volume (500 mL) of milk?

- Yes
- No

28.a. Would you be willing to drink this volume (500 mL) of milk after exercise?

- Yes
- No

**Page 8: Future Research**

29. Would you be willing to be contacted by our research group to participate in future research?

- Yes
- No

If you are willing to be contacted, please provide your preferred contact information below. You do not have to provide this information if you do not wish to be contacted.

30. If you are willing to be contacted, how would you prefer to be contacted? (Optional)

- Telephone
- Email
- Post

31. Full Name (Optional)

- (free-text response)

32. Contact details

32.a. Phone number (Optional)

Please enter a valid phone number.

- *(free-text response)*

32.b. Email address (Optional)

Please enter a valid email address.

- *(free-text response)*

32.c. Postal address (Optional)

- *(free-text response)*

**Page 9: Survey Feedback**

33. Please provide any feedback you have for this survey (Optional)

- *(free-text response)*

**Page 10: Final page**

Thank you for completing this survey! Your responses will help us to understand older adults' perceptions of muscle damage and exercise recovery. We hope to use this information to inform future recommendations for exercise and recovery strategies that may prevent muscle loss in older age.

If you have any queries regarding this research please email

e.j.hayes2@newcastle.ac.uk

Thank you again
