# Supplementary material for: Older Adults’ Knowledge and Perceptions of Whole Foods as an Exercise Recovery Strategy
Source: Front Nutr. 2021 Oct 4;8:748882. doi: 10.3389/fnut.2021.748882 (PMC8520979; doi:10.3389/fnut.2021.748882)
Supplement: Supplementary file 2 [file Table_4.DOCX]

**Supplementary Materials Appendix B**

**Table 5.** Older adults’ views on milk as an exercise recovery supplement^1^

| Code | n | Examples |
| --- | --- | --- |
| Acceptable |  |  |
| Like milk | 44 | - I believe that a glass of milk after exercise would be quite refreshing |
|  |  | - I like all dairy products and would find it easy to drink more milk |
| Believe in milk’s benefits | 40 | - Milk is a protein and would help building the damaged muscle |
|  |  | - As ex dairy farmer's wife and mother have appreciation of qualities in milk |
| Drink milk already | 17 | - I drink quite a lot of milk anyway. |
|  |  | - For many years I have consumed full fat, Blue Top milk at home. Three + Pints a week. |
| Natural | 13 | - Because it is a natural product |
|  |  | - I would prefer a healthy drink to drink containing artificial supplements. |
| Cheap/ accessible | 7 | - Milk is readily available |
|  |  | - Cheap and easy |
| Willing to try | 7 | - I don’t have a problem with milk, I would try it as a recovery method, and ditch it if it didn’t help |
| Other | 7 |  |
| May Be Acceptable |  |  |
| Dislike of milk | 25 | - I only drink milk in tea or coffee, or in yogurt form. I don’t like it neat much but would drink if I knew it would help. |
| Not heard of milk as a recovery aid | 20 | - I have never heard of milk being recommended |
|  |  | - Don’t know sufficient about it to give any other answer |
| Unconvinced of efficacy | 11 | - If you could show me the science behind this I would be willing to try. |
|  |  | - Tried it makes no difference |
| Drink milk already | 7 | - I take 200ml of milk as part of my daily intake |
|  |  | - Always consumed full fat milk and have tended to eat more cream in old age. |
| Wary of fat/cholesterol | 4 | - Don't drink too much as puts weight on. Always drink semi-skimmed |
| Like milk | 3 | - I could easily drink more milk. Easiest type of supplement I could use |
| Medical | 2 | - I only use milk in tea/coffee and use oat milk otherwise. I have a slight allergy to cow’s milk. I have asthma if I drink too much |
|  |  | - As type 1 diabetic milk has to be part of carbohydrate intake |
| Natural | 2 | - Milk is natural |
| Other | 17 |  |
| Unacceptable |  |  |
| Dislike of milk | 47 | - Dislike of the taste of milk on its own |
|  |  | - I cannot drink milk, as it makes me feel sick, but I do consume it in other ways, e.g. puddings. |
| Intolerance/allergy | 17 | - I am lactose intolerant |
|  |  | - My body makes too much calcium so have to be careful how much I take in and I don't like milk by itself |
| Belief that it is not needed | 9 | - I already have a good diet and my exercise routine has not suffered for not drinking milk! |
| Ethical concerns | 3 | - Cruelty of farming animals |
| Weight concerns | 2 | - Extra calories, mucus producing, difficult to transport to outside classes |
| Other | 2 |  |

^1^We are specifically interested in milk as an exercise recovery beverage in older adults. Would this be an acceptable strategy to you? Please explain your answer. (291 responses)
